# Effects of Probabilistic Risk Situation Awareness Tool (RSAT) on Aeronautical Weather-Hazard Decision Making

**DOI:** 10.3389/fpsyg.2020.566780

**Published:** 2020-12-16

**Authors:** Sweta Parmar, Rickey P. Thomas

**Affiliations:** Decision Processes Lab, School of Psychology, Georgia Institute of Technology, Atlanta, GA, United States

**Keywords:** NEXRAD, cumulative risk, risk situation awareness (RSA), weather-hazards, decision-making, probabilistic estimates, uncertainty

## Abstract

We argue that providing cumulative risk as an estimate of the uncertainty in dynamically changing risky environments can help decision-makers meet mission-critical goals. Specifically, we constructed a simplified aviation-like weather decision-making task incorporating Next-Generation Radar (NEXRAD) images of convective weather. NEXRAD radar images provide information about geographically referenced precipitation. NEXRAD radar images are used by both pilots and laypeople to support decision-making about the level of risk posed by future weather-hazard movements. Using NEXRAD, people and professionals have to infer the uncertainty in the meteorological information to understand current hazards and extrapolate future conditions. Recent advancements in meteorology modeling afford the possibility of providing uncertainty information concerning hazardous weather for the current flight. Although there are systematic biases that plague people’s use of uncertainty information, there is evidence that presenting forecast uncertainty can improve weather-related decision-making. The current study augments NEXRAD by providing flight-path risk, referred to as the Risk Situational Awareness Tool (RSAT). RSAT provides the probability that a route will come within 20 NMI radius (FAA recommended safety distance) of hazardous weather within the next 45 min of flight. The study evaluates four NEXRAD displays integrated with RSAT, providing varying levels of support. The “no” support condition has no RSAT (the NEXRAD only condition). The “baseline” support condition employs an RSAT whose accuracy is consistent with current capability in meteorological modeling. The “moderate” support condition applies an RSAT whose accuracy is likely at the top of what is achievable in meteorology in the near future. The “high” support condition provides a level of support that is likely unachievable in an aviation weather decision-making context without considerable technological innovation. The results indicate that the operators relied on the RSAT and improved their performance as a consequence. We discuss the implications of the findings for the safe introduction of probabilistic tools in future general aviation cockpits and other dynamic decision-making contexts. Moreover, we discuss how the results contribute to research in the fields of dynamic risk and uncertainty, risk situation awareness, cumulative risk, and risk communication.

## Introduction

We argue that cumulative risk is a critical piece of information to provide decision-makers because it can be coupled to action, supports what-if simulation, and can reflect the likelihood that a decision leads to failure of mission-critical goals or regulatory constraints. We briefly review the psychological literature concerning decision-makers’ understanding of risk and, in particular, cumulative risk. Our interpretation of the research leads us to argue that accurate subjective estimation of cumulative risk is likely impossible for humans to accomplish unaided. Moreover, expecting operators to estimate cumulative risk in decision contexts characterized by strong temporal dynamics, stress, and high workload seems unreasonable and illustrates the need to provide model-derived estimates of cumulative risk. After reviewing the risk communication literature to identify best practices for communicating cumulative risk, we then review the aeronautics literature as our application domain. We argue that many of the errors made by pilots in hazardous weather operations result from misunderstandings of the risks posed by hazardous weather and that providing operators forecast-derived cumulative risk estimates could support decision-making in such contexts. Finally, we report an original study whose results indicate that cumulative risk estimates support novices’ decision-making in a simulated aviation-like navigation task through convective weather.

Environmental and temporal dynamics are responsible for much of the uncertainty associated with decision making in many domains (e.g., tsunamis, hurricanes, earthquakes, aviation accidents, infectious diseases, and terrorist attacks). Natural dynamic situations contain much uncertainty as the dynamic circumstances continually change, leaving the decision-maker to consider the implications of the current situation and prospective temporal changes. Due in part to the uncertainty of a continuously evolving environment, trade-offs must exist between the cost of action and the risk of non-action (Kerstholt, 1994) have to be balanced. In these decision contexts, the demand for the tasks will often exceed operators’ available mental resources (Dörner and Pfeifer, 1993; Kunreuther et al., 2002; Kowalski-Trakofler et al., 2003). For this reason, the decision-maker needs to maintain an optimal level of situation awareness (see Endsley, 1995) about the uncertainty and risk associated with that uncertainty. However, risk situation awareness (RSA) is still not a well-studied topic or even a clearly-defined concept in the situation awareness literature. Although there is literature concerning how people incorporate available uncertainty information into their decisions, there is relatively little work assessing risk situation awareness (RSA) in emergency decisions under spatially-temporally distributed uncertainty.

It is essential to study how people perceive and assess risk; however, it is perhaps more important to identify what characteristics of risk need to be communicated for decision-makers to understand risk in a particular situation and make an informed decision. One crucial aspect of risk, and an often neglected one, is its cumulative nature. Extremely small risk levels in the near term can accumulate to become extremely hazardous in the long run when continuous exposure to the same risk factors occurs for a long duration (e.g., smoking for years) or simultaneous exposure to multiple factors (e.g., smoking and drinking). Perceptions of cumulative risk are studied widely in various socially significant issues like smoking (Weinstein, 1998; Slovic, 2000), climate change (Crawford-Brown and Crawford-Brown, 2012), contraceptive failures (Doyle, 1997), floods (De La Maza et al., 2019), Sexually Transmitted Diseases (Knauper et al., 2005), stroke risk (Fuller et al., 2004), medical treatments (Dijkstra et al., 2000), and so on. Most of these risky catastrophic events have a very low probability of happening in a particular month or a year; however, those probabilities can aggregate to a very high cumulative risk over a decade or lifetime, causing significant losses (Slovic et al., 1978). Although the chance of injury is 1:10,000 each time one drives, the lifetime chance is 1:3.

Even the simplest forms of the cumulative probability of encountering an event X at least once in T years, as calculated by Equation 1 below, is relatively complex and non-intuitive for humans (De La Maza et al., 2019).

(1) $$P\left(X\geq 1\right)=\sum_{t=1}^{T}p\times {\left(1-p\right)}^{t-1}=1-{\left(1-p\right)}^{T}$$

p = annual probability of an event.

t = index for each time period.

People tend to focus on (relatively small) one-time risks and repeated safe experiences, leading to underestimation of the cumulative risk posed by rare threats (Slovic, 1987; Slovic et al., 2007). Although calibrating one’s judgment and choices to the actual cumulative risk is challenging, it is practically impossible in situations with dynamic hazards. Risks in dynamic decisions can accumulate in relatively short, near tactical, timescales due to the presence of multiple hazardous events that are spatially distributed, temporally dynamic, that are regularly exhibiting some form of interdependence. An excellent example of a dynamic risk is severe storms where the movement of hazardous storm cells are not independent of one another in the airspace. The laws of fluid dynamics govern the movement of those red cells over relatively short time intervals. The weather has a similar significant influence in the navigation process for UAVs, submarines, road vehicles, aircraft, etc. Hence, the uncertainty associated with spatial-temporal hazards makes the computation of cumulative risk extremely difficult. Thus, models and data science applications are necessary to estimate these mission-centric risks for specific decisions.

We argue that the disjunctive form of cumulative risk is potentially beneficial for communicating risk in dynamic decision-making environments. We contend that the disjunctive form of cumulative risk should support operators’ decision-making to minimize exposure to even a single threatening event (e.g., avoiding a route that intersects any red-cell activity or hazardous weather even once). Moreover, the cumulative risk can reflect a mission objective like avoiding all convective weather by 20 NMI or staying out of the operable range of enemy assets in military operations.

An essential aspect of having models of mission-centric risk outcomes is understanding how people use the models’ probabilities to calibrate their decision making to the uncertainty. Unfortunately, it is important to acknowledge that behavioral decision theory has documented numerous systematic biases in how humans process uncertainty information (Gilovich et al., 2002). Gibbons et al. (2013) reported that although expert National Airspace System (NAS) operators understand that weather forecasts have errors and are uncertain, the operators are biased in their interpretations of weather information uncertainty (i.e., the probabilities). In other words, even when the operators in the Gibbons et al. (2013) study were aware of weather uncertainty, they were unable to account for the uncertainty appropriately in their decision-making. Thus, instead of assuming that decision makers can mentally compute cumulative risk via such a formula as presented via Eq. 1, research shows that people often engage in heuristic shortcuts (Juslin et al., 2015). Juslin et al. (2015) show that people often misinterpret cumulative risk and rely on additive and multiplicative heuristics when reasoning about cumulative risk. Apart from being prone to heuristics and biases, peoples’ understanding of cumulative risk is prone to framing effects (Doyle, 1997). Overall, it is well-established in the literature that cumulative risk is sensitive to multiple cognitive factors like motivation, heuristics, biases, risk information presentation formats, framing effects, etc. Hence, the need for models to estimate cumulative risk directly quantifying uncertainty in the system is necessary to improve RSA of operators in hazardous and dynamically evolving situations.

Even if models successfully calculate cumulative risk, there is still the challenge of communicating cumulative risk in a format that operators can understand to calibrate their decision-making. Doyle (1997) found that understanding of cumulative risk improved when framed as a disjunctive probability (the probability that an event will happen at least once), instead of a conjunctive probability (the probability that an event will never happen). Hence, providing some evidence representing uncertainty in terms of disjunctive probability can help calibrate people’s decisions to uncertainty in the system. We adopt the disjunctive framing of the cumulative risk in our implementation of communicating risk in the experiment.

Adopting a disjunctive frame leads to the choice of communicating risk in a graphical or numerical format. Numerical formats of risk are often shunned because of the extensive literature showing people have great difficulty understanding numerical risks. However, graphical/visual representations also have drawbacks. Some work has documented arbitrary cartographic design features (e.g., different color-schemes for coding probabilities) can influence how people assess the risk posed by hazardous weather (Klockow-Mcclain et al., 2020). Moreover, it seems that numerical formats of risk should be preferred given their precision if people can understand them. Fortunately, several studies have demonstrated that providing numerical forecast uncertainty information can increase the understanding of weather forecasts, which leads to better calibrated and more beneficial decisions for both expert meteorologists and laypeople (Joslyn and Nichols, 2009; Joslyn et al., 2009; Nadav-Greenberg and Joslyn, 2009; Joslyn and LeClerc, 2012; LeClerc and Joslyn, 2015). Joslyn and LeClerc (2013) argue a need to provide numerical estimates to support optimal decision-making in weather situations, where operators are already aware of the uncertainty but ill-equipped to calculate exact estimates. Thus, we chose to investigate the viability of a numerical format for communicating cumulative risk in our study.

Adopting a numerical representation for communicating risk leads to the critical choice between choosing a probability and a relative frequency format. Joslyn and Nichols (2009) address the long-believed notion in the decision-making literature that lay people find probability formats challenging to understand and account for in their decisions compared to frequency formats. Joslyn and Nichols (2009) tested the effects of giving uncertainty information in both probability (90% chance) and frequency (9 out of 10 chance) formats for daily wind speed forecasts. They found that, contrary to previous research, the information presented in frequency formats was the most difficult for people to utilize to understand the wind speed warnings. Frequency formats led to the largest number of errors in both people’s decision-making and their understanding of uncertainty. They observed that people were better able to understand wind speed forecasts with the probabilistic format and made better decisions. Based on Joslyn and colleagues’ work, we adopt a probabilistic format for communicating cumulative risk in our experiment.

Nadav-Greenberg and Joslyn (2009) demonstrated that repeated exposure to weather uncertainty information via training followed by feedback helps people learn to use the available uncertainty information for their decisions. We agree with prior researchers that trial-based training and immediate feedback about the numerical probability estimates’ accuracy are critical to learning. Thus, we implement such training and feedback to support the understanding of disjunctive risk in our experiment.

We focus on aviation-like navigation because the risk weather poses to a flight path is continually changing, making it practically impossible for pilots to calculate such risks on their own without sophisticated meteorological (probabilistic forecast) modeling. Our lab-based experimental task was a simplified version of a General Aviation (GA) pilot route-evaluation task. The experimental task is most analogous to en-route flight operations taking place in the vicinity of convective weather hazards under instrument meteorological conditions (IMC). In layperson terms, the task has some similarity to the types of evaluations that pilots might make circumnavigating thunderstorms at night. Thus, our experiment uses weather-related decision-making in an aviation-like context as our test case for investigating the use of cumulative risk in a dynamic decision-making task. Specifically, we investigated how well people can use mission-centric cumulative risk estimates of uncertainty to make flight-route evaluations.

According to the National Transportation Safety Board (NTSB, 2014), hazardous weather is a significant contributor to general aviation accidents, identified as a factor in 35% of all fatal accidents from 1982 to 2013 (Fultz and Ashley, 2016). Specifically, the two most common factors associated with such accidents are non-adherence of pilots to the FAA recommended minimum separation distance of 20 NMI from hazardous weather (FAASTeam, 2008; FAA, 2013), and the tendency of pilots to select risky routes for circumnavigating convective weather (Boyd, 2017). This risk underestimation by pilots results from the high level of complexity involved in weather-related decision-making and associated workload. Operations in inclement weather require pilots to make multiple evaluations and decisions, including identification of the presence of a hazard, estimation of the proximity of weather, estimation of hazard’s impact to the flight path, and take appropriate actions (Burgess and Thomas, 2004; Elgin and Thomas, 2004). The weather-related information that the pilot receives comes from many sources, including meteorological briefings, inflight weather reports, visual information from the cockpit, and on-site reports (Hunter et al., 2003). Because of the dynamic environment of the aircraft cockpit and the weather itself, data change very quickly. Pilots have to monitor for changes and update their mental representation of the situation accordingly to maintain both situation awareness (SA) and risk situation awareness (RSA).

Research shows that even after pilots receive information that signals a need to revise their plan, they often continue to follow the original flight plan (Orasanu et al., 2001). This tendency, known as plan continuation errors, could theoretically be mitigated by presenting continuously updated cumulative risk information in real-time for the flight plan and alternative projected paths. This argument seems plausible, particularly since plan continuation errors are most common with pilots who tend to inadequately monitor their airspace (Muthard and Wickens, 2002). Plan continuation errors are costly in the real world. They often happen when pilots decide to continue on a flight path, constructed under Visual Flight Rules, that they should divert as weather conditions deteriorate to IMC (Wiegmann et al., 2002). Providing cumulative risk estimates could also help alleviate some of the unwarranted optimism about the weather situation observed in pilots, leading them to continue flying through hazardous weather for extended amounts of time before making the correct decision to divert (Wiegmann et al., 2002).

One of the primary and ubiquitous cockpit weather information products used by pilots is NEXRAD. NEXRAD provides geographically referenced precipitation activity uplinked to cockpits through Flight Information Services Data Link (FISDL) (Wu et al., 2010). NEXRAD mosaic images provide cues to storm movement, and the reflectivity color-coding can be considered a rough proxy of the risk posed by weather hazards. Unfortunately, to our knowledge, all studies reported in the scientific literature using both professional and student pilots indicate that a substantial number of them approach weather hazards dangerously close, often penetrating red-cell activity (in simulation), or grossly overestimate their distance from the weather hazards (Novacek et al., 2001; Yuchnovicz et al., 2001; Beringer and Ball, 2004; Burgess and Thomas, 2004; Lemos and Chamberlain, 2004; Wu et al., 2011, 2012, 2013; ATSC, 2013). Delayed weather information is one of the primary contributors to navigation decision error (Latorella and Chamberlain, 2002). Hua et al. (2015) found that student pilots were prone to error projecting future storm positions from delayed weather radar information, even when the pilots had NEXRAD Looping. Although there has been significant progress addressing the latency and error in NEXRAD (Rude et al., 2012), there is still the problem that there will always be some latency in NEXRAD composite images due to the physical limitations of the radar and the time it takes to update the base data, generate and transfer the composite radar images to the cockpit (Elgin and Thomas, 2004; ATSC, 2013). Again, we argue that the provisioning of model-derived cumulative risk estimates could account automatically for the uncertainty imposed by the temporal delay and the effects of projected weather cell movements on the cumulative risk to the flight path. In current operations, pilots have to make these estimations unaided and likely under a high workload. Our experiment provides NEXRAD images to the participants that are precisely 15 min old with zero latency to simplify the task.

We are not the only researchers to argue and conduct research on the idea that one way to support pilot inferences about the uncertainty in weather-aviation products is to leverage probabilistic forecasts that explicitly render the uncertainty. Rockwell Collins’ Enhanced Weather Radar (EWxR) system combined NEXRAD and onboard radar to overcome the attenuation and range limitations of the onboard radar (Kronfeld, 2003). It also characterized the cells as hazardous, possibly-hazardous, or non-hazardous based upon attributes like reflectivity level, storm speed, and height. Therefore, the EWxR attempts to represent uncertainty by using the term “possibly hazardous”; however, the extent of uncertainty in quantitative format was missing, and the understanding of uncertainty depends on how the operator interprets the risk and uncertainty associated with the term “possibly hazardous.” Similar work was conducted by Spirkovska and Lodha (2002) when they provided go or no-go deterministic decisions about the current flight route to reduce plan continuation errors. Busquets et al. (2005) provided alert notifications for impending hazards in the pilots’ current flight route; however, without any risk information related to uncertainties associated with those hazards. Matthews and DeLaura (2010) developed the Convective Weather Avoidance Model (CWAM) that provided both deterministic and probabilistic weather avoidance fields (WAFs); however, the pilots in the study underestimated the risk associated with WAFs and intersected WAFs even when the probabilistic information was provided. All these attempts show that there is a need for more similar efforts to what Joslyn, Doyle, and others mentioned previously did in presenting clear uncertainty and risk estimates in a quantitative format.

Many state-of-the-art weather forecasting products and prototypes are being developed by the NCAR (National Center for Atmospheric Research) and UCAR (University Corporation for Atmospheric Research). Table 1 describes several relevant weather-forecast products for convection, icing, turbulence, and some of these products provide forecast uncertainty. Most notably, the National Convective Weather Forecast-2 (NCWF-2) provides probabilistic hazard fields for severe convective activity. The development of probabilistic hazard fields is an important advancement. However, probabilistic hazard fields might be interpreted in a deterministic manner and suffer similar misuse by pilots as the polygon projections of the original NCWF or the probabilistic WAFs (Burgess and Thomas, 2004; Matthews and DeLaura, 2010). The operator or pilot would also be responsible for estimating the flight-path disjunctive risk from the probabilistic hazard fields; and, as we reviewed above, pilots likely cannot do this unaided. Also, it is important to study how accurate probabilistic estimates need to be for effective use. This paper attempts to do that by investigating the effects of disjunctive risk on decision-making. One of the significant limitations of current aviation meteorological tools is that they do not render mission-centric risk (e.g., flight-path risk). Thus, we explore the potential benefit of providing cumulative flight-path risk in a direct numerical format to evaluate if this supports decision making to avoid dynamic hazards.

**TABLE 1:** Descriptions of several meteorological aviation-weather products.

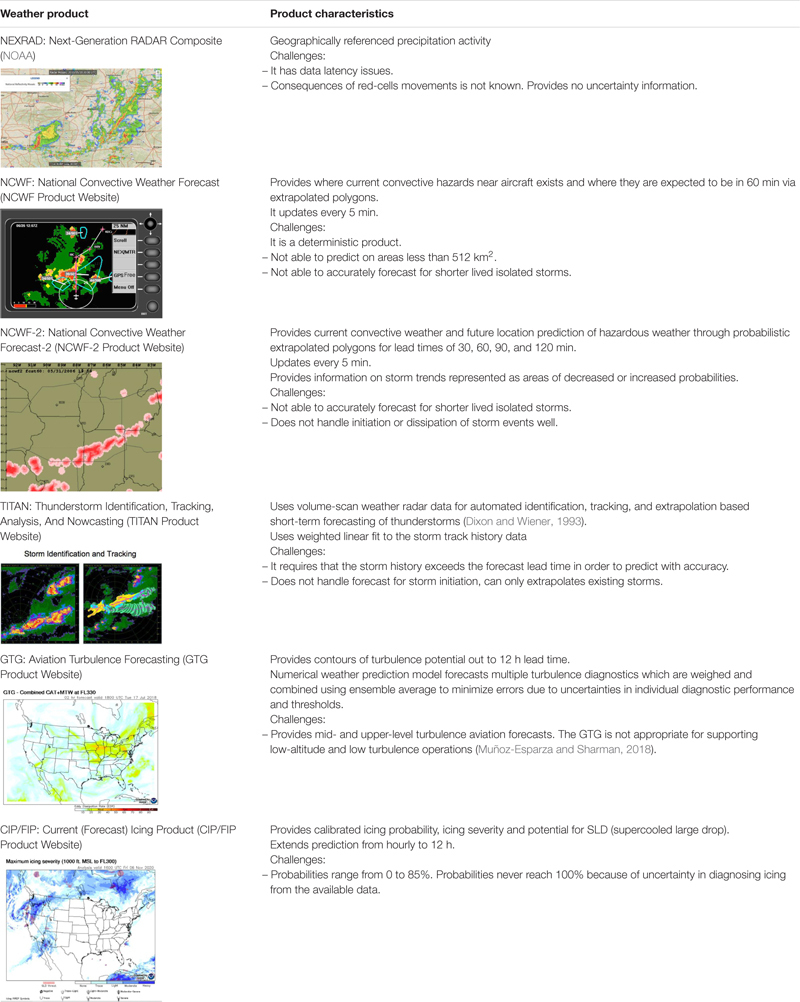

Specifically, the experiment evaluates a probability-based Risk Situation Awareness Tool (RSAT). RSAT is integrated with NEXRAD and provides a quantitative estimate (cumulative disjunctive risk) of the probability that hazardous weather will impact the current projected flight path within the FAA’s 20 NMI safety margin. This study’s primary objective is to evaluate whether the probabilistic RSAT improves the decision-making and uncertainty comprehension of the operators. The study also assessed the extent to which training and experience with the RSAT facilitate performance to a more ecologically-valid transfer task. Another goal was to determine the accuracy required for the probabilistic RSAT to support operator performance in terms of decision-making and calibration.

The hypotheses for the experiment are listed below. In general, the hypotheses reflect predictions that the disjunctive form of cumulative risk will support operators’ evaluations of flight-path risk.

• Hypothesis 1: The participants receiving RSAT will make more accurate flight-path decisions (high proportion correct and high sensitivity) and confidence judgments (low Brier score) in order of the level of accuracy of the RSAT. The high accuracy RSAT will lead to better-calibrated participant decisions to the system, compared to the participants receiving lower support.
• Hypothesis 2: The accuracy of the participants’ flight-path decisions and confidence judgments will improve with training. We also predict that learning will transfer from training to the test phase.
• Hypothesis 3: Performance will improve across training blocks as participants receive feedback and gain experience throughout the training phase.
• Hypothesis 4: Performance will improve across storms as participants receive feedback and gain experience throughout the test phase.
• Hypothesis 5: A storm’s unfolding behavior will improve the participants’ performance on later (within-storm) trials. We predict that the participants will utilize emergent cues to storm movement and make better flight-path decisions as the storm moves (on later trials).
• Hypothesis 6: Performance on double-route trials (relative judgment task) will be higher than performance on single-route trials (the absolute judgment task) for all four groups. This prediction is based on the findings from the area of sensation and perception, which shows that people find it easier to make judgments on relative tasks compared to absolute tasks (Weber and Johnson, 2009).
• Hypothesis 7: The participants’ trust in the RSAT will change depending on the manipulated accuracy of the RSAT. We expect that trust will be positively correlated with the level of support.

## Materials and Methods

### Participants

Three-hundred and forty-three Georgia Tech undergraduate students participated in this study. The participants were recruited via an online experiment management system, and they received course credits for their participation. Participants provided informed consent before the experiment. Participation in this experiment was voluntary and in agreement with the guidelines of the Institute Review Board (IRB). Only Three-hundred and twenty-four participants were included in the data analysis. All the participants with available response data for more than 85% of the test trials were included in the analysis.

### Design and Materials

**FIGURE 1:**
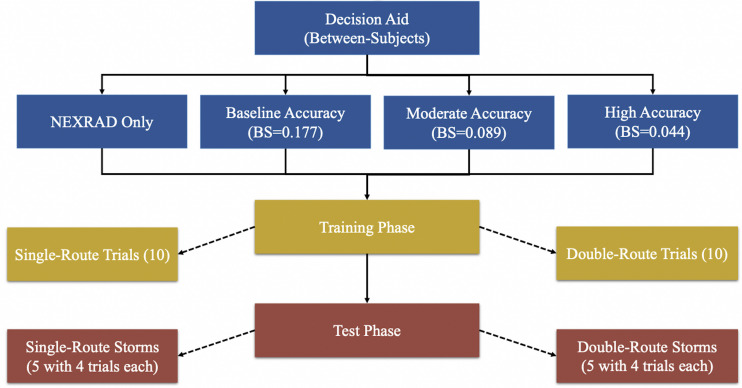
Overview of experimental design.

The study was designed using Qualtrics and PsychoPy (Peirce et al., 2019) software for participants to make weather-related flight-path safety decisions. The experiment design (Figure 1) was a 2 (trial type) × 2 (experiment phase) × 4 (RSAT) mixed design. Both the trial type and the experiment phase manipulations were within-subject factors, and the RSAT manipulation was a between-subjects factor. The RSAT manipulation had four levels: (1) no support (NEXRAD only), (2) low support (NEXRAD + baseline-accuracy RSAT), (3) moderate support (NEXRAD + moderate-accuracy RSAT), and (4) high support (NEXRAD + high-accuracy RSAT). The trial type manipulation had two levels: (1) a single route trial (absolute judgment), and (2) a double route trial (relative judgment). The experiment phase manipulation had two levels: (1) the training phase, and (2) the test phase. The study had the following dependent variables: performance (proportion of correct weather-related decisions), trust score [trust in automation scale by Jian et al. (2000)], calibration (Brier score and Brier skill score), sensitivity and bias (signal detection theory). Participants were assigned randomly to the four between-subject conditions, N (No support condition) = 73, N (Baseline support condition) = 81, N (Moderate support condition) = 88, and N (High support condition) = 82.

The probabilistic Risk Situation Awareness Tool (RSAT) was simulated to produce probabilities for the forecasts from the available historical hazardous storm data NEXRAD images extracted from (NOAA). To support replication of the experiment, we include all NEXRAD images, storm descriptions, flight paths, and RSAT probability for each condition on a publicly accessible repository (DropBox Repository). The RSAT rendered flight-path cumulative risk as a probability that the projected flight path will come within 20 NMI radius (FAA recommended safety distance) of hazardous weather within the next 45 min of flight. Half of the routes were constructed to intersect hazardous weather in the next 45-min of sustained flight, and the other half were constructed to be “safe.” The route generation was not automated. Routes were manually constructed to meet several criteria: no routes had red-cell activity rendered in the starting range ring, for the double-route trials- one route was always “safe,” and the other intersected hazardous weather. All projected flight paths were superimposed on each NEXRAD at a length of 40.28 statute miles (35 nautical miles), reflecting every 15 min (one flight segment) of flight time at 140 knots. We also counterbalanced the labels (A&B) to “safe” and “unsafe” routes such that the labels were not indicative of outcomes to avoid biasing evaluations. All the trials were created using different, extremely hazardous storms in the United States—it would be unsafe to penetrate these storms, given the significant amount of red reflectivity and high echo tops at the ceiling of most general aviation aircraft. We intentionally chose such powerful storms to simplify the task, so routing (vectoring) was the only viable escape maneuver. The range ring aided these judgments and provided a symbolic representation of the 20 NMI FAA safety recommendation.

The outcome index of the routes (scored 0s if safe and 1s if they intersected weather) were used to generate probabilities for the RSAT that reflected different levels of pre-specified aggregate accuracy as measured via the Brier Score (BS) (Brier, 1950; Murphy and Winkler, 1977; Yates, 1990). In other words, we generated probabilities that conformed to different target BS values for each support condition. The RSAT disjunctive probabilities were simulated such that the Brier Score of the baseline accuracy RSAT was 0.17 [level of accuracy reflecting current meteorological capabilities (ATSC, 2015)], moderate accuracy RSAT was 0.09 (level of accuracy achievable with some technological innovation), and high accuracy RSAT was 0.04 (level of accuracy that could only be achieved in aviation weather context with considerable technological advancement). Note that the probabilities were generated with knowledge of the outcome of a route, but not the characteristics of the storm or the rendering of the flight-path itself. Basically, the probabilities were randomly assigned to routes respecting only the outcome index. To be clear, the only difference between the support conditions is the accuracy of the probabilities displayed next to the flight routes. In other words, in the high-support condition, probabilities (flight-risk estimates) were closer, on average, to 100% for routes that would intersect weather and closer to 0%, on average, for routes that would not intersect weather compared to the lesser support conditions. For example, in the moderate-support condition a Brier score of 0.09 implies a 70% (30%) probability will be presented on average for routes that ultimately intersect (not intersect) weather hazard. In comparison, in the high-support condition, a Brier score of 0.04 implies an 80% (20%) probability, on average, will be presented for routes that will intersect (not intersect) weather hazard. Again, RSAT probabilities for each condition are available on a publicly accessible repository (DropBox Repository).

**FIGURE 2:**
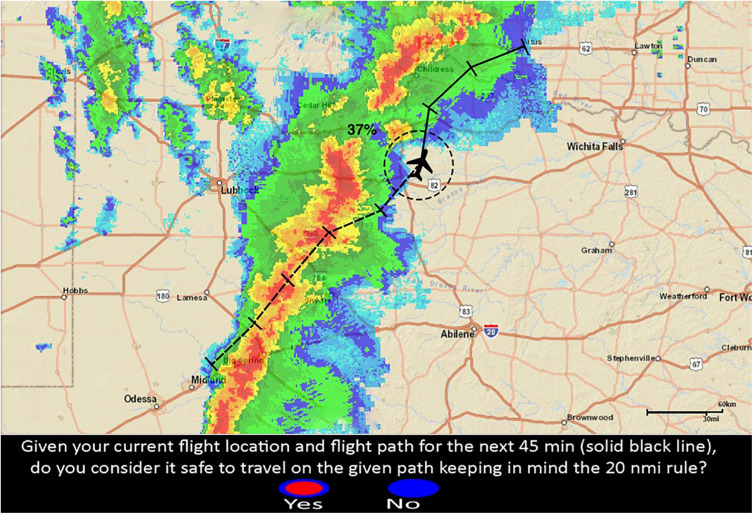
Single route trial with RSAT. The disjunctive risk (37%) is located next to the ownship icon and represents the probability that at least one red-cell is forecasted to intersect the projected flight-path in the next 45 min of flight (the solid line).

**FIGURE 3:**
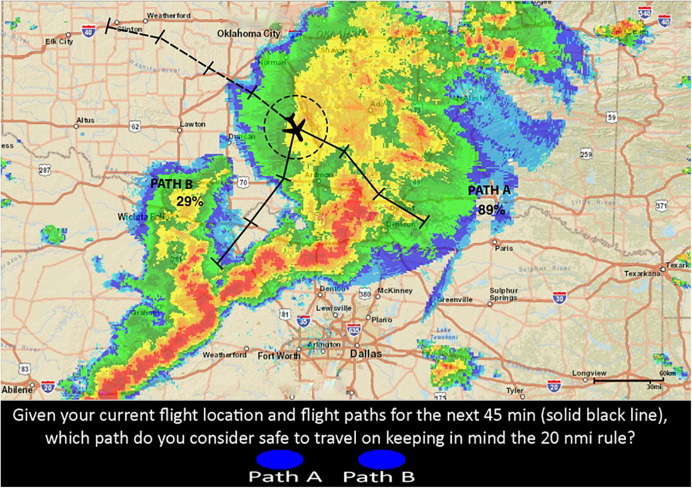
Double route trial with RSAT. The disjunctive risk estimates are located near their corresponding paths and are 89% for Path A and 29% for Path B. The disjunctive risk represents the probability that at least one red-cell is forecasted to intersect each projected flight-path in the next 45 min of flight (the solid line).

Figure 2 represents a single-route decision trial. Figure 3 represents double-route decision trial providing a probability of 89% for path A and 29% for path B. Meaning that path A has an 89% chance of intersecting the red cells in the 20 NMI range ring (at least once) during the 45 min projected route, and path B has a 29% chance of intersecting the red cells (at least once). Ideally, if the RSAT was deterministic, the participant should always choose to select the route with the lower probability. However, it is impossible to design a completely accurate system for hazardous storms due to uncertainty. So, people need to account for this uncertainty in RSAT while using it to inform their situation awareness about risk. The same Brier Score will be used to measure Confidence Judgment (CJ) calibration.

### Procedure

Participants were presented with a simulated flight path in an adverse weather condition, and they had to decide whether the route was safe to continue flying based on the FAA’s 20-NMI rule. Again, Figures 2, 3 represent examples of two different decision trials and the corresponding decision that the participant had to make. The 20 NMI-radius circle (range-ring) on the flight route represents the safe area around the current flight location, which served as a guide for participants to keep clear of any red weather cells. The solid black line on the route was the projected 45 min future flight path for which participants had to make safety-decisions. The black ticks represent 15 min time intervals for the given route. The dashed line represents the aircraft’s previous flight path. All of the display features mentioned above were present for all participants on every trial. Participants also were to assume constant altitude (cruising altitude) and constant speed (140 knots) for the entire experiment. It is important to acknowledge that real pilots want to know their altitude and consider escape maneuvers via altitude. However, we intentionally selected powerful thunderstorms from the archival record that were not escapable via altitude changes (high echo tops) and where it wouldn’t be advisable to approach the storm at a flight level with low reflectivity due to the potential for (low or no reflectivity) turbulence. We suspect that storms this powerful could generate air turbulence 3,000–5,000 ft from red-cell reflectivity.

**FIGURE 4:**
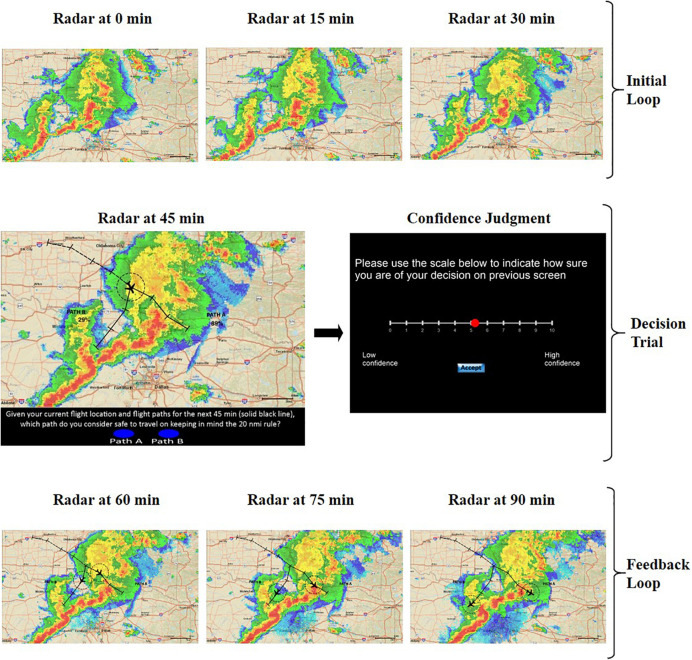
Elicitation for double route trial in the training phase: Initial NEXRAD loop followed by decision trial and confidence judgment, and final feedback loop.

**FIGURE 5:**
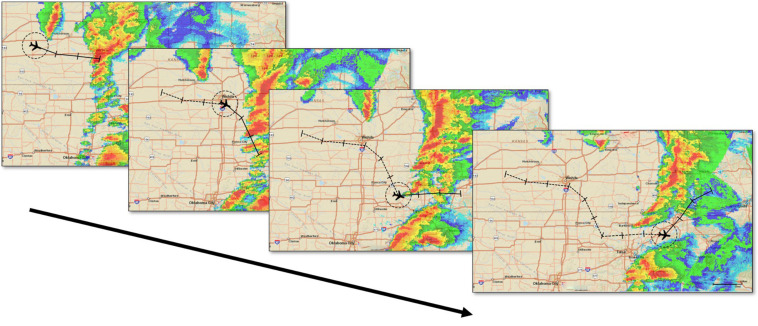
Elicitation for unfolding trials in the test phase. The four NEXRAD images depict four 45-min segments for a 3-h flight.

**FIGURE 6:**
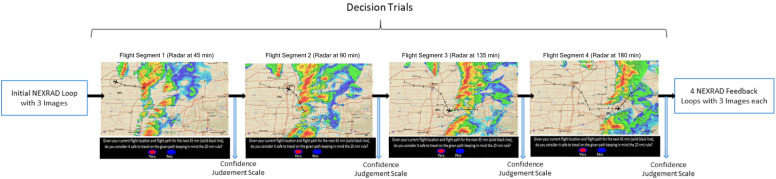
Flow diagram of test phase trials.

The experiment consisted of a training phase followed by a test phase (See DropBox Repository for all trial images). Both the experiment phases had single and double route trials randomly presented. The operators rated their confidence for every flight-path decision immediately after they made that decision. The training phase had 20 trials with both single and double route trials in equal proportion, presented at random. All the trials of the training phase were extracted from a different hazardous scenario (or storm). Figure 4 shows an example trial of the training phase. Before every decision trial, three NEXRAD images for every 15 min interval before the decision time were looped once, providing the participants with the 45-min historical weather data. Following every decision trial, participants rated their confidence in their flight-path decisions. Immediate feedback was provided by three NEXRAD images (looped once) for every 15 min interval after the decision time point, indicating whether the flight-path intersected a weather hazard within the 20 NMI range ring. The test phase presented 10 different storms with four unfolding trials each (Figures 5, 6) presented at random. One initial NEXRAD loop, similar to Figure 4, was presented before the first trial of each storm, and the feedback loop (for all four unfolding decisions) was shown after the fourth trial. The test phase evaluated whether the learning on the probabilistic RSAT and/or NEXRAD from the training phase transferred to the more ecologically-valid test-phase task (unfolding storm trials).

The goal was to evaluate whether the RSAT support significantly improves upon the no support condition in terms of both decision-making performance and calibration to the uncertainty in the weather situation. Moreover, we expected performance to improve with the increased accuracy of the probabilities presented across the different RSAT conditions (baseline, moderate, and high). All the trials were the same in all four conditions. The only difference between conditions was the absence or presence of the disjunctive probability values and their accuracy. It was feasible to simulate the probabilities from the accuracy (BS) benchmark because radar images from the past hazardous storms were publicly available, so those storms’ future outcome was known (via the available radar record of the entire storm event). Also, we did not provide any explicit information regarding the accuracy of the NEXRAD display to the participants because such information can distort use and expectations (Barg-Walkow and Rogers, 2016). We wanted to investigate the effects of experiential learning as cleanly as possible. Thus, we expected the participants to learn the display’s accuracy from the feedback provided to them. We evaluate learning in the four conditions via the participants’ bias and sensitivity scores across the RSAT trials. The transfer of learning is through the relationship between training performance and test performance.

### Dependent Variables

The dependent measures were sensitivity and bias (signal detection theory), Brier and Brier skill scores (calibration), the trust in automation scale, and the proportion correct (accuracy).

#### Calibration

The most common metric for the analysis of confidence judgment is the probability score known as the Brier Score (Brier, 1950; Murphy and Winkler, 1977; Yates, 1990). The Brier score (BS) is a proper scoring rule that provides a measure of the accuracy of confidence judgments:

(2) $$B\,S=\frac{1}{N}\,\sum_{T=1}^{N}{\left(c_{t}-o_{t}\right)}^{2}$$

The Brier score is described by Eq. 2, where *N* is the total number of probability or confidence assessments, *c*_t_ is the *t*^th^ confidence judgment, and *o*_t_ is the outcome index for the *t*^th^ confidence judgment. If the event occurs, then *o*_t_ = 1, and if the event does not occur, then *o*_t_ = 0. Thus, the Brier score is the average squared deviation between the confidence of the decision-maker and the outcome index (Brier, 1950; Murphy and Winkler, 1977; Yates, 1990). The lower the Brier score for a set of predictions, the better their calibration (i.e., less error).

Another measure, the Brier Skill Score (BSS), acts as an overall measure of the system’s performance (Wilks, 1995). It measures the relative skill of a forecast above a reference, the *BS*_*ref*_ term in Eq. 3. We adopted the baseline or control group BS as the reference forecast accuracy.

(3) $$B\,S\,S=\frac{\left(1-B\,S\right)}{B\,S_{r\,e\,f}}$$

The BSS ranges from minus infinity to 1. BSS = 0 implies no skill compared to the reference forecast. BSS = 1 is a perfect score. Hence, BS is a measure of the accuracy of predictions or forecasts. BSS is a measure of the proportion of improvement in accuracy over the reference (Dance et al., 2010).

#### Performance

The proportion of correct decisions is used as a measure of the accuracy of simulated flight-path decisions. Of course, it is well-known that the response to a stimulus depends on the individual’s sensitivity to the stimulus and the individual’s decision criterion (or bias). Thus, in addition to simple proportion correct, we use measures of both sensitivity and bias to model decision making in our uncertain situation (Swets et al., 2000). Sensitivity or Discriminability Index (*d*′) measures the operators’ ability to tell that two signals are different. Response Bias or Criterion (C) reflects an individual’s implicit decision threshold above which they respond “yes” to the presence of a signal and below which they respond “no” to the presence of a signal.

#### Trust in Automation Scale

**FIGURE 7:**
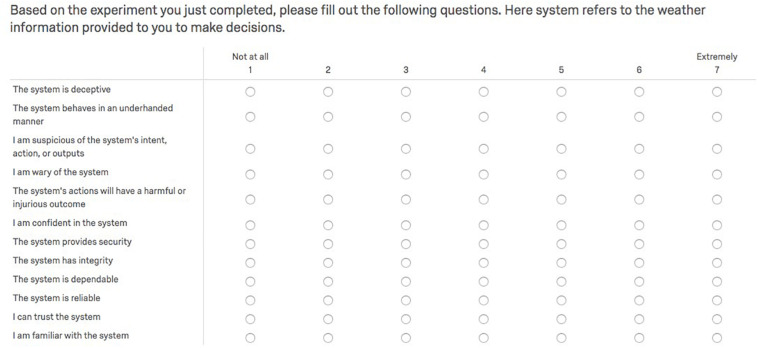
Trust in automation scale by Jian et al. (2000).

We measured if changes in trust in the tool were related to the manipulated accuracy of the provided disjunctive probabilities. At the end of the test phase of the experiment, participants rated the 12-item Trust in Automation scale (Figure 7), developed by Jian et al. (2000). It is one of the most used instruments in the literature to measure human trust in systems and equipment. The total score on the scale is indicative of the level of trust in the automation with high scores interpreted as overtrust in the system, and the lower scores interpreted as distrust in the system. We also consider the per trial confidence judgments as a surrogate measure of trust at the trial level of analysis. In the results section, we explore the relations between trust rating and subjective confidence.

### Statistical Analysis

The Generalized Estimating Equations (GEE) for categorical data was used to derive parameter estimates for both sensitivity and bias within a single model specification [via the use of a probit link function, DeCarlo (1998)]. Parameter estimates for each factor manipulation represents the changes in decision threshold (in beta units), and parameter estimates for each factor manipulation crossed with “truth,” the actual occurrence of the storm, represents changes in discriminability (in *d*′ units). The BS and BSS were analyzed using a GEE with an identity link function and normally distributed errors, because the scores were continuous. To assess learning, we analyzed proportion correct using a GEE with a probit link and binomial distribution because the percent correct is a binary variable. To evaluate learning transfer, we relied on both the parametric Pearson Correlation and the non-parametric Kendall’s Tau correlation for both performance (percent correct) and BS. The trust in automation scores was evaluated via one-way ANOVA, followed by *post hoc* comparisons and correlations.

## Results

### Sensitivity and Decision Bias

**FIGURE 8:**
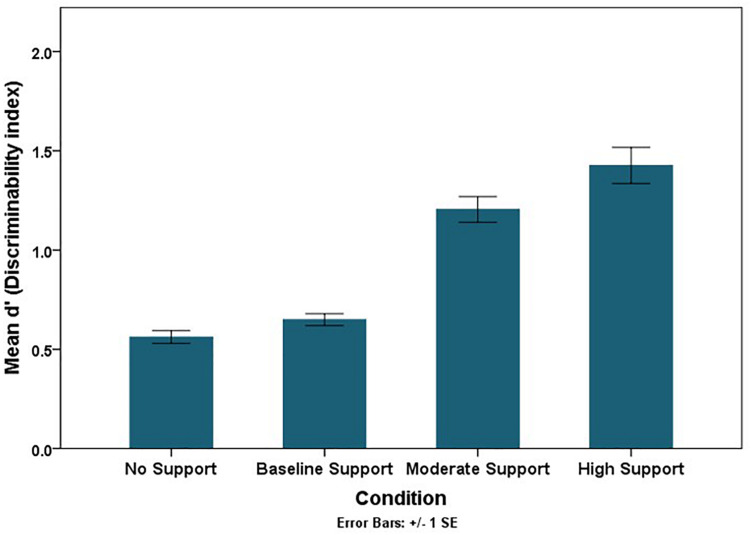
Mean discriminability trend across conditions.

**FIGURE 9:**
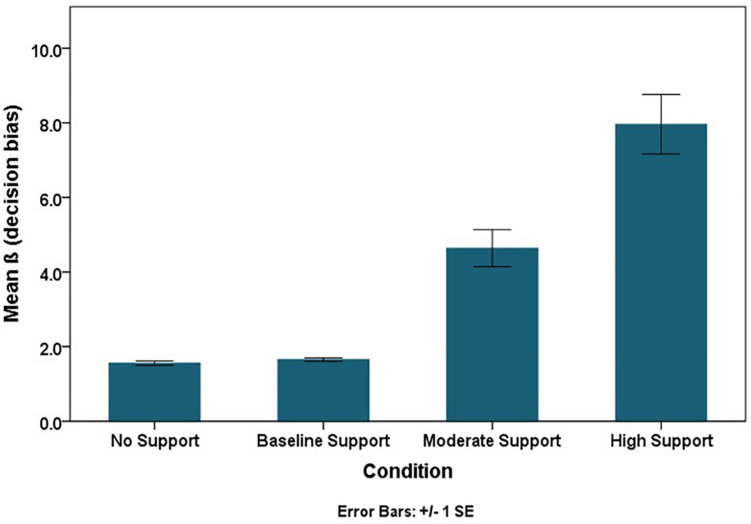
Mean bias trend across conditions.

The RSAT should facilitate people’s ability to discriminate if their flight path will intersect hazardous weather (sensitivity). Although it is important to separate accuracy or sensitivity from a support tool’s effects on people’s risk aversion (response thresholds), both aspects of decision-making are necessary to evaluate (Swets et al., 2000). The class of Generalized Estimating Equations (GEE) for categorical data can estimate sensitivity and bias parameters within a single model specification (via a probit link function). Using the probit link function, parameter estimates for each factor manipulation represents the changes in decision threshold (in beta units), and parameter estimates for each factor manipulation crossed with “truth,” the actual occurrence of the storm, represents changes in discriminability (in *d*′ units) (Table 2). Sensitivity or discriminability improved as the accuracy of the RSAT increased (the mean trend is illustrated in Figure 8). Discriminability improved (*p* < 0.0001) in the test phase compared to the training phase with the increase in the level of accuracy of the RSAT (Truth × Experiment Phase × Condition), and it also improved more in double route trials (*p* < 0.01) compared to single route trials with the increase in the accuracy of the RSAT (Truth × Trial Type × Condition). The decision thresholds significantly increased as the level of support increased (Figure 9). This finding is consistent with the idea that the decision-makers adopted more conservative thresholds as the accuracy of the RSAT increased.

**TABLE 2:** Summary statistics for Type 3 generalized estimating equations analysis, displaying flight-path safety decisions regressed on information characteristics and task outcome.

Independent variable	Df	Wald Chi-square statistic
RSAT condition	3	9.30*
Trial type	1	13.51***
Truth	1	945.16****
Experiment phase	1	45.74****
RSAT condition × Trial type	3	7.91
RSAT condition × Truth	3	89.14****
RSAT condition × Experiment phase	3	5.68
Trial type × Truth	1	106.70****
Trial type × Experiment phase	1	104.18****
Truth × Experiment phase	1	89.49****
RSAT condition × Trial type × Truth	3	15.32**
RSAT condition × Trial type × Experiment phase	3	15.49**
RSAT condition × Truth × Experiment phase	3	32.46****
Trial type × Truth × Experiment phase	1	3.24
RSAT condition × Trial type × Truth × Experiment phase	3	4.29

*p < 0.05, **p < 0.01, ***p < 0.001, ****p < 0.0001.

### Confidence Judgment Calibration

Brier scores (BS) were used to evaluate the calibration of confidence judgments. The lower the BS, the better calibrated the confidence judgments. There was a significant decrease in the mean Brier Score as the accuracy of the RSAT increased, χ^2^(3) = 90.73, *p* < 0.0001 (Table 3). Moreover, the Brier Scores were significantly lower in the relative judgment trials compared to the absolute judgment trials, χ^2^(1) = 77.99, *p* < 0.0001, and in the test phase, compared to the training phase, χ^2^(1) = 108.87, *p* < 0.0001. Although the participants’ calibration increased as the accuracy of the RSAT increased, the mean BS was still lower than the corresponding BS for the RSAT itself (Table 3). Thus, the participants were less calibrated than the RSAT and failed to exploit all the information it provided. Another important observation to note was that the mean BS for NEXRAD-only condition was approximately equal to mean BS of 0.25 for chance performance. We should note that the high-support condition did show a minor deviation in kurtosis from normality (Table 4). The statistical inferences and the interpretations of the results were unchanged after reanalyzing with a square root transformation of the BS.

**TABLE 3:** Group-wise mean brier score and brier skill scores.

RSAT condition	Mean BS for RSAT	Mean BS for participants	Mean BS for participants		Mean BSS for participants (ref-no support)
			Training phase	Test phase	
No support	–	0.24	0.26	0.24	–
Baseline support	0.18	0.22	0.25	0.22	0.08
Moderate support	0.09	0.18	0.21	0.16	0.26
High support	0.04	0.14	0.18	0.12	0.41

**TABLE 4:** Brier Score descriptive statistics (standard error in parentheses).

	No support	Baseline support	Moderate support	High support
Mean	0.24 (0.005)	0.22 (0.004)	0.18 (0.008)	0.14 (0.01)
Median	0.24	0.22	0.18	0.15
Variance	0.002	0.002	0.006	0.008
Skewness	0.71 (0.28)	0.37 (0.27)	0.15 (0.26)	0.151 (0.27)
Kurtosis	0.52 (0.56)	0.45 (0.53)	−0.742 (0.51)	−1.32 (0.53)

We also calculated the Brier Skill Score (BSS) to scale the improvement in accuracy provided by the RSAT compared to the control (NEXRAD only) condition. Referring to Table 3, the higher the BSS for the support conditions, the better the calibration was over the control condition.

### Learning Effects

#### Transfer of Learning

The training phase’s participant performance predicted the test phase’s performance (Table 5), indicating the transfer of learning. Performance in Table 5 is the mean proportion of correct decisions. Interestingly, the transfer (τ_b_) increased with the increase in the level of support, except for the non-significant transfer of learning for the no support condition. A similar pattern emerged for the Brier Scores, indicating the transfer of calibration (Table 5). For both Brier Score and performance, the strength of the correlation increased with the increase in the level of support, indicating that probabilistic support facilitated transfer to a more ecologically-valid task environment.

**TABLE 5:** Kendall’s Tau and Pearson’s correlations [τ_b_ (*r*)] matrix between training and test phase.

	Test phase performance				
	No support	Baseline support	Moderate support	High support	Total
Training phase performance	0.14 (0.16)	0.20** (0.29)	0.47**** (0.61)	0.66**** (0.77)	0.45**** (0.61)
	**Test phase brier score**				
Training phase brier score	0.25** (0.38)	0.26**** (38)	0.53**** (0.69)	0.60**** (0.76)	0.48**** (0.71)

** p < 0.01; **** p < 0.0001.

#### Training Phase Learning

**FIGURE 10:**
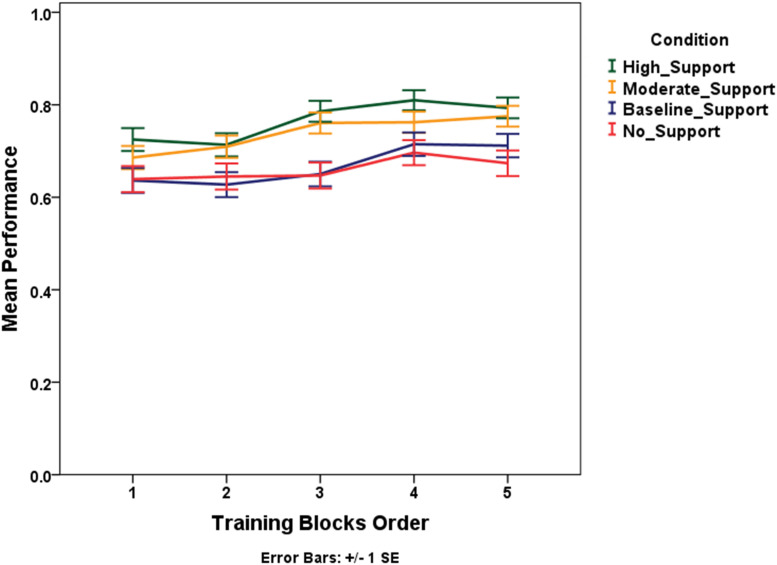
Mean performance (proportion correct) trend for subsequent training blocks across conditions.

We employed a generalized linear model for repeated measures to test for performance differences across training blocks, conditions, and scenarios. We included a scenario factor in the model for all the within-subject learning because the hazardous storms varied in strength, movement, uncertainty, and, ultimately, difficulty. The block factor divided the 20 trials of the training phase into five sets of 4 trials each in the order they were presented. The analysis indicates that the main effect of training blocks is significant, i.e., performance significantly increased across training blocks, χ^2^(4) = 24.36, *p* < 0.0001 (Figure 10). Thus, providing support for learning within the training phase. There is no significant interaction between condition and training blocks, χ^2^(12) = 13.24, *p* > 0.05, indicating that the performance over subsequent training blocks doesn’t change significantly with the change in the level of support.

#### Test Phase Learning

**FIGURE 11:**
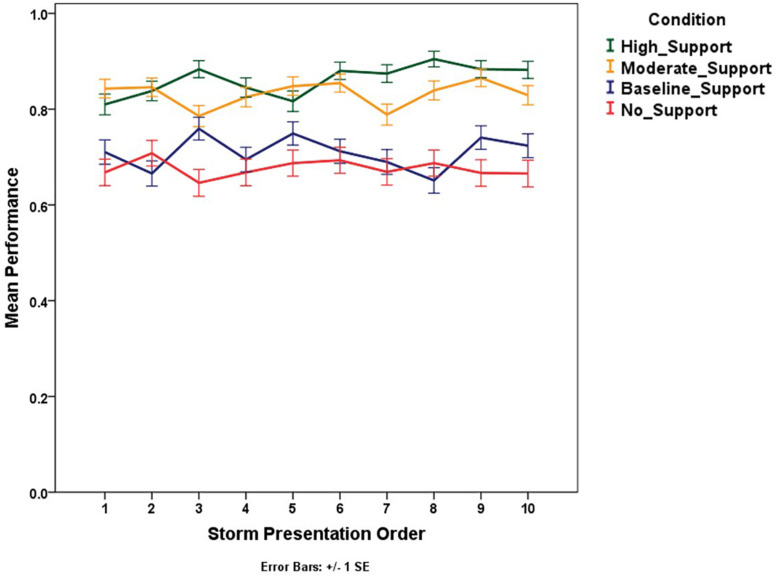
Mean performance (proportion correct) trend for subsequent storms presented across conditions.

In the test phase, each scenario had four different unfolding trials in which the same storm was evolving in time. To address our predictions regarding learning within the test phase, we employed a Generalized Linear Model for repeated measures to test the differences in performance between conditions, storm presentation order, unfolding trial order, and scenario. The main effect of storm presentation order is significant, χ^2^(9) = 18.24, *p* < 0.05, providing additional support for learning (Figure 11). Moreover, the interaction between condition and storm presentation order is also significant, χ^2^(27) = 51.6, *p* < 0.01, indicating that performance improved within the test phase and improved more for the high support condition than the other support levels.

**FIGURE 12:**
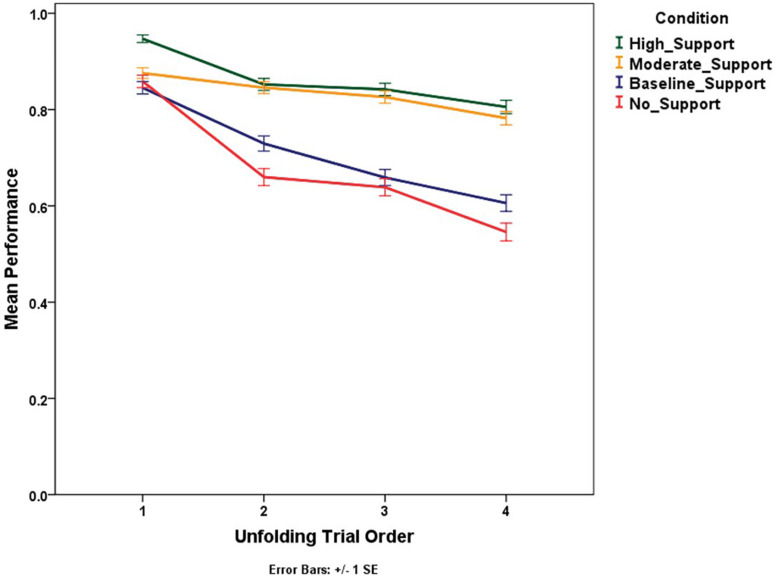
Mean performance (proportion correct) trend for unfolding trials across conditions.

The test phase’s performance for unfolding trials was expected to increase from the first trial to the fourth due to familiarity with storm movement. However, the performance was found to decrease with the unfolding storm trials consistently, χ^2^(3) = 183.78, *p* < 0.0001 (Figure 12). This might be due to the information available from the initial NEXRAD loop, which became increasingly old as the trials unfolded.

### Trust in Automation

Twenty-Seven participants failed to click the link that directed them to the trust scale at the end of the experiment. The omnibus trust score was calculated by reverse scoring the first five items in the trust scale (distrust questions) and then taking an average of all 12 responses (Figure 7). Researchers have reported that responses on the Jian et al. (2000) trust scale suffer from positive skew (Gutzwiller et al., 2019); however, the trust scores collected in this experiment did not exhibit skewness inconsistent with normality. Moreover, the trust ratings conformed to normality and variance assumptions of ANOVA (Table 6). A one-way ANOVA indicated a significant mean difference in trust score across support level, *F*(3,293) = 6.52, and *p* = 0.0001 < 0.05. Tukey’s HSD test for *post hoc* comparisons to test where the mean difference existed only indicated significant mean differences in trust scores between the baseline support (*M* = 4.02, *SD* = 0.91) and the moderate support (*M* = 4.57, *SD* = 0.93) conditions, *p* = 0.003 < 0.008 and the baseline (*M* = 4.02, *SD* = 0.91) and the high support (*M* = 4.58, *SD* = 1.07) conditions, *p* = 0.003 < 0.008. Hence, there was only partial evidence for the trust score predictions that the trust scores will increase with the increase in the level of accuracy of RSAT.

**TABLE 6:** Trust and component trust scores descriptives statistics (standard error in parentheses).

	Mean	Median	Variance	Skewness	Kurtosis
**Omnibus trust score**					
No support	4.16 (0.11)	4.20	0.85	−0.22 (0.29)	−0.66 (0.57)
Baseline support	4.02 (0.10)	4.00	0.83	−0.08 (0.27)	−0.99 (0.53)
Moderate support	4.57 (0.11)	4.67	0.87	−0.58 (0.27)	−0.08 (0.54)
High support	4.58 (0.13)	4.71	1.15	−0.51 (0.29)	−0.70 (0.56)
**Component trust score**					
No support	4.27 (0.12)	4.43	0.90	−0.29 (0.29)	−0.79 (0.57)
Baseline support	4.07 (0.11)	4.00	1.02	−0.15 (0.27)	−0.92 (0.54)
Moderate support	4.70 (0.12)	4.86	1.06	−0.76 (0.27)	−0.11 (0.54)
High support	4.66 (0.14)	4.93	1.38	−0.70 (0.29)	−0.61 (0.57)

We suspect that the trust items in the automation scale (Figure 7) did not fit the context of a probabilistic RSAT. The distrust items seem a particularly ill-fit to the RSAT context, and the distrust items also suffered significantly more attrition (46–69% response rate) than the trust items (81–91% response rate). We decided to replicate the omnibus trust score analysis using only the trust items to address the missingness problem. The analysis of the mean score of the trust items met ANOVA’s assumptions (Table 6), and the statistical inferences and pattern of findings were unchanged from the omnibus trust score results. A one-way ANOVA indicated a significant mean difference in component trust score across support level, *F*(3,290) = 6.54 A one-way ANOVA indicated a significant mean difference in trust score across support level, *F*(3,293) = 6.52, and *p* = 0.0001 < 0.05. Tukey’s HSD test for *post hoc* comparisons to test where the mean difference existed indicated significant mean differences in component trust scores between the baseline support (*M* = 4.07, *SD* = 1.01) and the moderate support (*M* = 4.70, *SD* = 1.03) conditions, *p* = 0.001 < 0.008 and the baseline (*M* = 4.07, *SD* = 1.01) and the high support (*M* = 4.66, *SD* = 1.17) conditions, *p* = 0.004 < 0.008.

Because one could argue that Confidence Judgment (CJ) and Brier Score (BS) are logical surrogates of trust (reliance) in the system at the trial level of analysis, we decided to conduct *ad hoc* analyses evaluating the relationship between the trust scale items and calibration (Table 7). The overall trust score and the component trust score obtained from the trust items were significantly correlated with CJ (*p* < 0.0001) and BS (*p* < 0.0001) in the expected direction for only the high support condition (Table 7). A similar trend was found for all 7-individual trust items—six out of the seven trust items were significantly correlated with BS and CJ for only the high support condition, and most of the correlations for the other support conditions were not significant. The component distrust score (from five items) and five individual distrust items were not correlated with CJ and BS except for a small significant correlation between the BS and the component distrust score (for high support), the BS and the wary item (for no support), and the BS and the deceptive system item (for moderate support) (see Supplementary Materials for item-wise correlations).

**TABLE 7:** Kendall’s Tau and Pearson’s correlations [τ_b_ (*r*)] correlations matrix for Trust scores.

	No support		Baseline support		Moderate support		High support	
	CJ	BS	CJ	BS	CJ	BS	CJ	BS
Overall trust score	0.12 (0.15)	−0.04(0.03)	0.02 (0.02)	−0.11(−0.16)	0.10 (0.08)	−0.16(−0.26*)	0.29****(0.34)	−0.36****(−0.53)
Component distrust score	−0.01(−0.05)	0.05 (0.04)	0.001(−0.004)	0.1 (0.17)	−0.04(−0.05)	0.14(0.25*)	−0.17(−0.19)	0.27**(0.39)
Component trust score	0.16 (0.21)	0.05 (0.09)	0.05 (0.08)	−0.12(−0.17)	0.05 (0.03)	−0.11(−0.19)	0.30****(0.38)	−0.40****(−0.55)

*p < 0.05, **p < 0.01, ****p < 0.0001.

## Discussion

The results indicate that operators did rely on the flight-path RSAT to improve their performance over the no-support condition. The Brier Skill Score for the high support condition in reference to the no support condition was 0.41. Thus, operator performance improved in terms of both calibration and resolution as the accuracy of the tool increased. However, the results suggest caution as performance is relatively low in conditions most similar to the current capability of meteorological forecasts (no support, BSS = 0, or baseline support, BSS = 0.07). Therefore, suggesting a need for similar research with pilots followed by the validation of training regimes for the safe introduction of high-accuracy probabilistic tools in general aviation cockpits.

A myriad of performance and calibration measures show consistent and statistically reliable differences between the levels of support. However, the calibration level (to the uncertainty) reached by the operators was still well below the tool’s calibration accuracy (Table 3), so there is further room for improvement. It could be that an alternative format of uncertainty (probability) presentation or explicitly telling operators the expected accuracy of the tools could lead to more reliance on the tool and a better understanding of the uncertainty (Barg-Walkow and Rogers, 2016). As we discuss next, training and instruction on these tools is necessary for people to utilize them to their full potential as cumulative risk and probability are not intuitive concepts. The literature we reviewed suggest that professionals often have trouble understanding cumulative risk, and we suspect that pilots will require training to calibrate their weather-hazard decision-making to uncertainty.

The substantial transfer of learning effects from the training phase to the test phase (unfolding storms) in the RSAT conditions implied that the participants understood and used the probabilistic rendering of risk. The training and test phase’s learning effects indicate the potential importance of enhancing learning via multiple trials- and scenario-based training. This finding has implications for implementing experience-based modules for many professional domains, including training pilots on probabilistic weather tools to improve their risk situation awareness.

We had predicted performance increments for flight-path decisions as a storm unfolded (i.e., unfolding trails), so we were quite surprised that there was a strong performance decrement trend. We interpret the performance decrement as an indicator that the NEXRAD initial loop after the first unfolding trial was important for operators to extract cues indicative of storm movement. Also, the absence of feedback compared to the training phase could have hindered the participant’s ability to update and correct their mental representation of storm movement and temporal dynamics. This finding also suggests that additional looping after every flight-path decision during the unfolding trial might have improved performance. These findings emphasize a need for continuous feedback and access to historical data to facilitate real-time decisions about hazardous weather and other dynamic risks. However, further research on the benefits of looping is needed. Several studies suggest that looping NEXRAD does not support pilots making safe flight decisions under hazardous weather conditions (Burgess and Thomas, 2004; Knecht, 2016a, b).

The operators’ trust scores didn’t reflect their performance or the quality of the RSAT provided to them. This finding is somewhat surprising given that the performance and calibration measures imply that the participants relied on the RSAT. Interestingly, the exploratory analysis showed that the tool’s trust level was only calibrated to the operator’s objective performance when the tool was highly accurate. Thus, the non-intuitive trend in trust scores might be attributed to the structure of the trust scale itself, which seems suitable for a higher level of automation or a warning system instead of a tool that renders uncertainty via numerical probability. Our results also indicate the possibility of people calibrating their trust in the system to their accuracy only when the system was highly accurate. It may be the case that some level of accuracy has to be achieved by operators for them to trust a system, even if the system’s accuracy is invariant. If true, this would account for the positive correlation between trust and calibration in the high-accuracy RSAT condition. In other words, relatively few participants seemed to achieve a level of accuracy necessary to trust the tool, suggesting the threshold for trust was most likely to manifest when the tool itself was highly accurate.

The fact that we utilized university students using a simplified task rather than expert pilots using higher-fidelity simulation is an important limitation of the study. Pilots receive training on weather decision-making (see U.S. Department of Transportation, 2016) and access many weather products in the cockpit when making real-world operational decisions. One of the benefits of using naïve or student-pilots as participants is that they might have fewer preconceived notions (both positive and negative) concerning forecasts’ performance. More realistic tasks have greater ecological validity, but it could overwhelm participants if they try to use all the weather information typically provided in the cockpit. Even expert pilots might find it challenging to use their typical products with the added workload of trying to learn and understand the cumulative risk probabilities. Under such conditions, participants might choose to ignore the probabilities in favor of more familiar products—everyone has seen NEXRAD images on their locale weather station. Acknowledging the students and task limitations, the study established that the participants utilized disjunctive risk estimates, and follow-up work with experts in higher-fidelity tasks seems prudent. We think that presenting cumulative risk estimates is one of the best options for minimizing the amount of weather information required in the cockpit.

Overall, the results of this paper have implications for new interventions in the aviation industry. It has implications for display designers in terms of introducing products with probabilistic weather information into the market place as well as introducing the regulatory regime for the entry of such products into the cockpit. These results guide the development of probabilistic tools that increases the risk situation awareness of operators. Thus, it addresses some of the issues faced by earlier attempts by Kronfeld (2003), Matthews and DeLaura (2010), and others in introducing qualitative probabilistic risk information in cockpits, which pilots misunderstood. The transfer of learning and within-phase learning can help develop and improve training programs for weather displays. These findings should reinforce the need for training to include dedicated modules for weather displays and the interpretation of probabilistic weather products, which is only a negligible portion of the training pilots receive currently.

The results also contribute to basic research in judgment and decision-making of how to facilitate people’s understanding of cumulative risk and uncertainty. The findings are relevant to the long-believed notion that people find probability formats too challenging to understand and incorporate into their decision-making (Tversky and Kahneman, 1974; Slovic, 1997). The results also suggest that participants did gain some understanding of the risk estimates in probability format and were able to calibrate their decision-making to the accuracy of the risk estimates. Hence, our research provided further evidence that people can use probability formats effectively, at least in some contexts [c.f., the work of Joslyn and Nichols (2009), who showed that non-experts could understand probabilistic rain forecasts].

The results also have implications for some of the challenges demonstrated in conveying cumulative risk in various domains like breast-cancer medication risk (Zikmund-Fisher et al., 2008), contraception methods related risk (De La Maza et al., 2019), stroke risk (Fuller et al., 2004), flood risk (De La Maza et al., 2019), etc. mentioned before. The results show that via scenario- and feedback-based training, it is possible to calibrate people’s judgment and decision-making to the numerical risk estimates and the inherent uncertainty in the complex mission-centric task of flight-route judgments. Thus, it seems reasonable that these findings can extend to domains with less complexity in which the events are independent and homogenous.

Model-derived risk estimates should, in theory, be useful to operators, particularly operators under time and workload stress. However, to make risk estimates useful, operators will need to understand how the estimates are derived and have some nominal level of trust in the provided cumulative probabilities to rely on them. As stated earlier, we believe that simulation-based experiential training could be a pivotal component to operators understanding uncertainty in their domain and ultimately gaining a level of familiarity with the system to engender trust. This type of simulation training might focus on emergencies because they should be relatively rare and unfamiliar situations to the operator operationally and are the conditions under which operators will undoubtedly experience a high workload (Baumgart et al., 2008). Another advantage of model-derived uncertainty is that it can be adapted to What-If simulations—the risk estimate could be tailored to the specific actions an operator is considering. Similarly, the cumulative risk posed to the mission goal could inform operators working in command and control settings. What-If capabilities should facilitate operator intuitions to the uncertainty inherent in the domain and their ability to assess risk. Such What-If tools should also facilitate trust because they can evaluate how well the tool matches the risk evaluation of their actions (e.g., the cumulative risks associated with operator-generated routes).

Future research could build upon this study’s findings by developing a suite of algorithms that leverage ensemble modeling utilizing archival big weather data to build a product capable of real-time cumulative risk estimates operationally. An obvious ecologically-valid extension of this study is the use of high-fidelity flight simulators and professional pilots to evaluate how the current findings extend to a more specific operational context. Methods to build operator trust in these kinds of probabilistic systems also should be explored. Although the trust items on Jian’s trust scale apply to predictive decision support systems, the distrust items don’t fit well conceptually, as indicated by the high attrition rate on those items. Thus, the trust in automation results highlights the need to develop and validate a trust in automation scale that is more applicable to predictive decision-support systems.

Another idea might be to investigate the RSAT as a discrete warning system instead of providing weather uncertainty as a metric appropriate for providing continuous support. Many domains have warning systems. For example, in aviation, there are warning systems for collision avoidance of terrain (TAWS), ground (GPWS), and traffic (TCAS). Maybe a hazardous weather system that relies on cumulative risk is a reasonable decision support technology to pursue.

Another limitation of the experiment is that we utilized NEXRAD images that were precisely 15 min old with no variance. Recent advances in the area of providing more timely radar data are exciting. The potential for gap-filling radars to support aviation weather products is important, as more precise and updated radar data supported meteorologist assessments and warning decisions (Rude et al., 2012). Another area of consequence for enhancing aviation weather products is CASA (Collaborative Adaptive Sensing of the Atmosphere) (Brotzge et al., 2010), which may mitigate some of the challenges that delayed weather information pose to pilots for navigating around convective weather. Radar composite image age and latency are challenges that should be addressed in future work.

Presenting cumulative risk estimates seems to have the potential to minimize weather information required in the cockpit and supporting weather-avoidance decisions. The relative success of the RSAT in a simplified weather-hazard situation could mean that similar metrics and tools could support situation awareness of cumulative risk in other dynamic decision-making domains characterized by strong temporal dynamics. Of course, the calculation of cumulative probability in most domains with strong temporal dynamics requires specialized models tailored to the distinctive dynamics and decision context. As such models advance, continued work on how best to interface the outputs with human operators will be necessary for safe adoption and deployment.

## Data Availability Statement

The raw data supporting the conclusions of this article will be made available by the authors, without undue reservation.

## Ethics Statement

The studies involving human participants were reviewed and approved by Georgia Institute of Technology Central Institutional Review Board. The patients/participants provided their written informed consent to participate in this study.

## Author Contributions

SP and RT conceived and planned the experiments. SP carried out the experiments. SP and RT contributed to the interpretation of the results. SP took the lead in writing the manuscript. Both authors provided critical feedback and helped shape the research, analysis and manuscript.

## Conflict of Interest

The authors declare that the research was conducted in the absence of any commercial or financial relationships that could be construed as a potential conflict of interest.
