# Supplementary material for: Effects of Probabilistic Risk Situation Awareness Tool (RSAT) on Aeronautical Weather-Hazard Decision Making
Source: Front Psychol. 2020 Dec 16;11:566780. doi: 10.3389/fpsyg.2020.566780 (PMC7772149; doi:10.3389/fpsyg.2020.566780)
Supplement: Supplementary file 1 [file Table_1.docx]

Supplementary Material

# Supplementary Table 1. Kendall’s Tau and Pearson’s correlations [τ_b_ (r)] matrix for trust scale items, and Confidence Judgments and Brier Scores

| **τ_b_ (r)** | **No Support** | | **Baseline Support** | | **Moderate Support** | | **High Support** | | **Total** | |
| --- | --- | --- | --- | --- | --- | --- | --- | --- | --- | --- |
|  | **CJ** | **BS** | **CJ** | **BS** | **CJ** | **BS** | **CJ** | **BS** | **CJ** | **BS** |
| **Deceptive System** | -.05  (-.13) | .09  (.10) | .05  (.06) | .12  (.18) | -.15  (-.19) | .23^*^  (.31) | -.02  (-.02) | .14  (.24) | -.06  (-.08) | .18  (.24) |
| **Underhanded** | -.04  (-.05) | .08  (.07) | .02  (.05) | .06  (.13) | .20  (.33^*^) | -.04  (-.07) | .04  (.05) | .09  (.18) | .03  (.08) | .07  (.08) |
| **Suspicious** | -.07  (-.15) | .08  (.15) | -.07  (-.05) | .15  (.18) | .14  (.21) | .19  (.23) | .17  (.22) | .08  (.07) | .01  (.03) | .13^*^  (.16) |
| **Wary** | -.24^*^  (-.27) | .04  (.01) | -.11  (-.13) | -.05  (-.10) | .09  (.22) | .06  (.05) | -.13  (-.21) | .20  (.24) | -.10  (-.11) | .08  (.13) |
| **Harmful** | .16  (.18) | .06  (.04) | -.02  (.03) | .06  (.11) | -.14  (-.07) | .24  (.21) | -.20  (-.35) | .36^**^  (.45) | -.06  (-.05) | .22^****^  (.27) |
| **Confident** | .23^**^  (.31) | -.001  (.04) | .00  (.005) | -.11  (-.13) | .13  (.08) | -.19^*^  (-.23) | .39^****^  (.46) | -.46^****^  (-.61) | 20^****^  (.23) | -.23^****^  (-.33) |
| **Security** | .17  (.18) | .06  (.15) | .02  (-.004) | -.10  (-.12) | .16  (.14) | -.17  (-.23) | .31^****^  (.40) | -.44^****^  (-.58) | .19^****^  (.22) | -.21^****^  (-.31) |
| **Integrity** | .08  (.10) | .22^*^  (.31) | .04  (-.003) | -.12  (-.15) | .07  (.09) | -.20  (-.16) | .24^**^  (.30) | -.36^****^  (-.47) | .14^**^  (.14) | -.15^**^  (-.21) |
| **Dependable** | .13  (.17) | .11  (.16) | -.04  (-.10) | -.002  (.04) | .20^*^  (.24) | -.15  (-.24) | .25^*^  (.31) | -.34^***^  (-.45) | .16^**^  (.18) | -.15^**^  (-.23) |
| **Reliable** | .14  (.21) | .04  (.06) | .10  (-.03) | -.02  (.01) | .00  (.09) | -.19^*^  (-.28) | .21^*^  (.26) | -.31^****^  (-.43) | .13^**^  (15) | -.18^****^  (-.25) |
| **Trust** | .15  (.14) | .01  (.08) | .04  (.06) | -.10  (-.12) | .16  (.16) | -.19  (-.20) | .26^**^  (.31) | -.34^****^  (-.47) | .18^****^  (.20) | -.22^****^  (-.30) |
| **Familiarity** | .02  (.08) | -.21^*^  (-.32) | .17^*^  (.24) | -.23^**^  (-.29) | -.04  (-.03) | -.08  (-.11) | .13  (.09) | -.17  (-.20) | .10^*^  (.13) | -.21^****^  (-.26) |

*p<0.05; **p<0.01; ***p<0.001; ****p<0.0001
